# Solvent minimization induces preferential orientation and crystal clustering in serial micro-crystallography on micro-meshes, *in situ* plates and on a movable crystal conveyor belt

**DOI:** 10.1107/S1600577514017731

**Published:** 2014-10-09

**Authors:** Alexei S. Soares, Jeffrey D. Mullen, Ruchi M. Parekh, Grace S. McCarthy, Christian G. Roessler, Rick Jackimowicz, John M. Skinner, Allen M. Orville, Marc Allaire, Robert M. Sweet

**Affiliations:** a Photon Sciences Directorate, Brookhaven National Laboratory, Upton, NY 11973, USA; b Office of Educational Programs, Brookhaven National Laboratory, Upton, NY 11973, USA; c Physics Department, University of Oregon, Eugene, OR 97403-1274, USA; d Suffolk County Community College, Selden, NY 11784, USA; e Biosciences Department, Brookhaven National Laboratory, Upton, NY 11973, USA

**Keywords:** *in situ* X-ray data collection, crystallography, acoustic droplet ejection, serial crystallography

## Abstract

X-ray diffraction data were obtained at the National Synchrotron Light Source from insulin and lysozyme crystals that were densely deposited on three types of surfaces suitable for serial micro-crystallography: MiTeGen MicroMeshes™, Greiner Bio-One Ltd *in situ* micro-plates, and a moving kapton crystal conveyor belt that is used to deliver crystals directly into the X-ray beam. 6° wedges of data were taken from ∼100 crystals mounted on each material, and these individual data sets were merged to form nine complete data sets (six from insulin crystals and three from lysozyme crystals). Insulin crystals have a parallelepiped habit with an extended flat face that preferentially aligned with the mounting surfaces, impacting the data collection strategy and the design of the serial crystallography apparatus. Lysozyme crystals had a cuboidal habit and showed no preferential orientation. Preferential orientation occluded regions of reciprocal space when the X-ray beam was incident normal to the data-collection medium surface, requiring a second pass of data collection with the apparatus inclined away from the orthogonal. In addition, crystals measuring less than 20 µm were observed to clump together into clusters of crystals. Clustering required that the X-ray beam be adjusted to match the crystal size to prevent overlapping diffraction patterns. No additional problems were encountered with the serial crystallography strategy of combining small randomly oriented wedges of data from a large number of specimens. High-quality data able to support a realistic molecular replacement solution were readily obtained from both crystal types using all three serial crystallography strategies.

## Abstract 2

Strategies are described for optimizing the signal-to-noise of diffraction data, and for combining data from multiple crystals. One challenge that must be overcome is the non-random orientation of crystals with respect to one another and with respect to the surface that supports them.

## 1. Introduction   

Serial micro-crystallography efforts currently explore diverse strategies for combining data from a large number of crystals that transit the X-ray beam either tumbling freely (Boutet & Robinson, 2008 ▶; Weierstall *et al.*, 2008 ▶) or deposited on a surface (Ji *et al.*, 2010 ▶; Soares *et al.*, 2011 ▶; Roessler *et al.*, 2013 ▶). New X-ray sources enable the use of micro-crystals because they can focus down to a few micrometers. In high-throughput fragment screening applications, serial crystal handling can accelerate data collection (Tsai *et al.*, 2013 ▶) and micro-crystals can accelerate fragment binding (Cole *et al.*, 2014 ▶). Challenging structure determination projects such as viruses, membrane proteins and protein–protein complexes will also benefit because these proteins often yield crystal showers. Radiation damage usually prevents obtaining a complete data set from a single micro-crystal, so contributions from many specimens must frequently be merged. At third-generation micro-beam facilities such as APS-GM/CA, diffraction decays to half-maximum (Henderson, 1990 ▶) in a few seconds; future synchrotron improvements will continue to push the capability for virtually instantaneous data collection. For example, the exposure limit under a 1 µm beam at the National Synchrotron Light Source II (NSLS II) FMX beamline is expected to be 5 ms (Hodgson *et al.*, 2009 ▶).

One ubiquitous feature affecting the ultimate viability of serial crystallography strategies is the minimum size that is desirable for specimens in real space and for wedges of data in reciprocal space. Diffracting power is directly proportional to crystal volume so that it is possible to determine the minimum feasible size [19.5 µm for insulin, 4:4:1 habit, 〈*I*/σ(*I*)〉_last shell_ = 2, 1.8 Å; Holton & Frankel, 2010 ▶] needed for a single specimen to yield full data before it is dimmed by radiation damage (Teng & Moffat, 2002 ▶). This minimum crystal size can be overcome if complete data is assembled from multiple crystals. However, merging a very large number of tiny data wedges presents its own challenges.

Most of the serial crystallography strategies that our group is currently exploring require that crystals be deposited onto a two-dimensional surface (flat micro-meshes, planar well bottoms on *in situ* micro-plates, and a horizontal conveyor belt). Crystal clustering problems can occur when crystals are deposited onto such flat data-collection media with minimal solvent (solvent minimization is required for optimal signal-to-noise). Electron microscopists are familiar with the tendency of small objects to cluster in thin water films due to surface tension, making it difficult to image each specimen individually. As very small protein crystals are increasingly used for X-ray crystallography it seems likely that they will aggregate in a similar way. Clean single-crystal diffraction data will require either probing the sample with a tiny beam that is small compared with most crystal-to-crystal distances, or preventing clustering using some physical method akin to electron microscopy grids (Kubalek *et al.*, 1991 ▶). Alternatively, software improvements may increase the robustness of data reduction to handle overlapping diffraction from crystal clusters. In addition to clustering, solvent minimization may induce crystals with flat sides to preferentially orient relative to the data-collection media, and data-collection strategies must be adjusted to measure all data.

There is a pressing need to determine the requirements of serial micro-crystallography efforts so that the characteristics of mini-beam and micro-beam X-ray facilities can be chosen to match. The Protein Crystallography Research Resource (PxRR) is operating the mini-beam facility at NSLS-X25 and simultaneously participating in the construction of two micro-beam facilities at NSLS-II. Faced with the inevitable trade-offs between different capabilities, we were motivated to begin finding answers to some of these questions. Our group has successfully demonstrated unique new approaches to serial micro-crystallography, such as acoustic droplet ejection (Ellson *et al.*, 2003 ▶), which uses sound waves to move crystals directly from their growth trays onto data-collection media (Soares *et al.*, 2011 ▶; Cuttitta *et al.*, 2014 ▶; Roessler *et al.*, 2013 ▶) or to grow protein crystals on plates (Villaseñor *et al.*, 2012 ▶) or directly on micro-meshes (Yin *et al.*, 2014 ▶). Initially, our serial crystallography efforts were frustrated by difficulties such as preferential orientation and crystal clustering. Here we explore the advantages and challenges of assembling a complete data set from a large number of micro-crystals that are serially presented to the X-ray beam on micro-meshes, in *in situ* micro-plates, and on a kapton crystal conveyor belt.

## 2. Methods   

**Figure 1:**
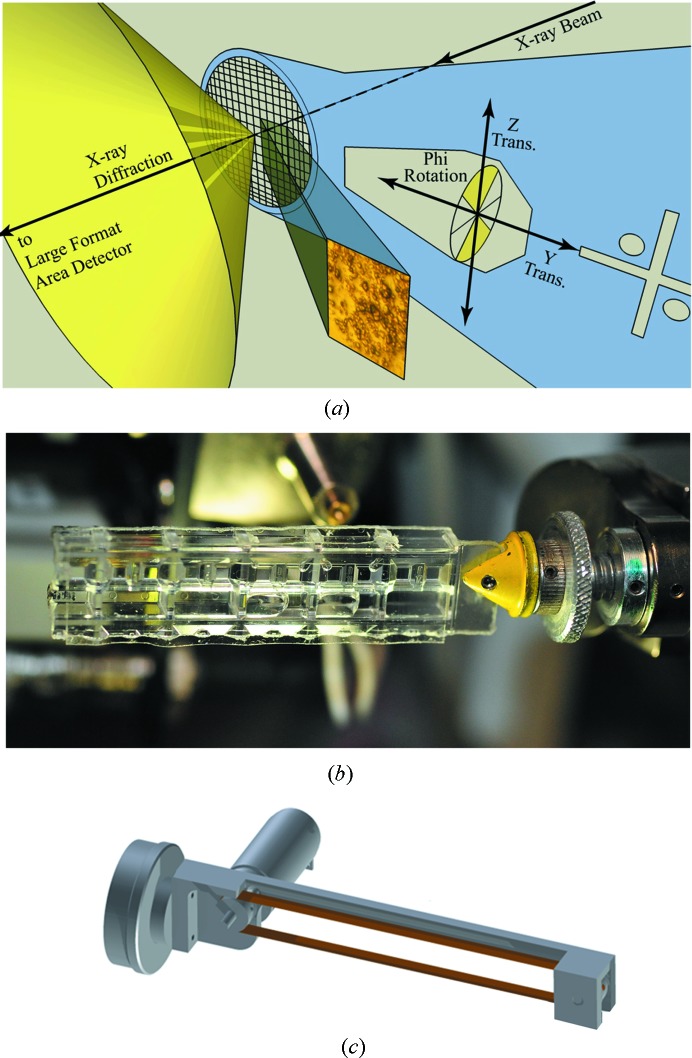
Data-collection strategy. The X-ray beam was used to scan for crystals using standard grid-scan techniques. When crystals were found, a 6° wedge of data was obtained (either a single 6° rotation or three 2° rotations). Serial data were obtained from crystals deposited on micro-meshes (*a*), on *in situ* micro-plates (*b*) and on a moving conveyor belt system (*c*) (Roessler *et al.*, 2013 ▶). In each case, diffraction from ∼100 individual crystals was collated into a high-quality data set. Data analysis was aided by triaging software. Initially, all data were obtained with the X-ray beam normal to the flat surface of the media on which crystals were deposited. Additional data were obtained with the X-ray beam tilted 30–60° relative to the flat crystal-containing surface (the additional data were needed because preferential orientation of the crystallographic *c* axis limited the maximum obtainable completeness using this strategy to ∼70%, as described in §3.2).

Serial crystallography is expected to generate a large volume of data that is best handled by data acquisition software that is closely integrated with a database and with image handling software. Our strategy was to identify crystals on flat data-collection media using standard X-ray grid scan techniques (Héroux *et al.*, 2014 ▶). Our grid scanning effort was deployed within our existing CBASS beamline control architecture, with built-in data triage using *distl* (Zhang *et al.*, 2006 ▶), data processing functionality using *EDNA* (Incardona *et al.*, 2009 ▶) and access to all output files and metadata *via* our PXDB database (Skinner *et al.*, 2006 ▶). Surfaces containing micro-crystals were oriented orthogonal to the X-ray beam. Beamline software automatically detected small crystals on micro-meshes using standard grid search techniques, and data were obtained with user-selected data collection parameters (Fig.1*a* ▶). *In situ* crystals in crystallization micro-plates (Fig.1*b* ▶) and crystals on the conveyor belt (Fig. 1 ▶ *c*) were large enough (50 µm) that grid scanning was not needed.

### 2.1. Specimen preparation   

The diffraction from very small crystals was easily overwhelmed by background noise when mother liquor was not carefully minimized. Most of the crystals were manually transferred onto MiTeGen micro-meshes (a few crystals were acoustically mounted; data not shown^1^). To improve the signal-to-noise and decrease the contribution of the mother liquor to the X-ray background, the solvent around manually mounted crystals was minimized in a humidity chamber using an absorbent material (such as a dental point) to dab dry each micro-mesh from the bottom. A video of this procedure is available through our YouTube channel (http://www.youtube.com/channel/UCtCiMjlzBnq5VYZzrEi3EiQ). To demonstrate the importance of solvent minimization, we used MicroRT™ X-ray Capillaries (MiTeGen) to obtain room-temperature X-ray diffraction data from the same thermolysin crystal both before and after solvent minimization. The capillary sheath was then removed and the crystal was cooled in liquid nitrogen for X-ray data acquisition at cryogenic temperatures. Thermolysin was used for this experiment because it crystallizes into a rod-shaped habit that is readily partitioned into three X-ray targets (one before solvent minimization, one after solvent minimization, and one at 100 K). The data were greatly improved by the solvent minimization procedure (see §3.1).

**Figure 2:**
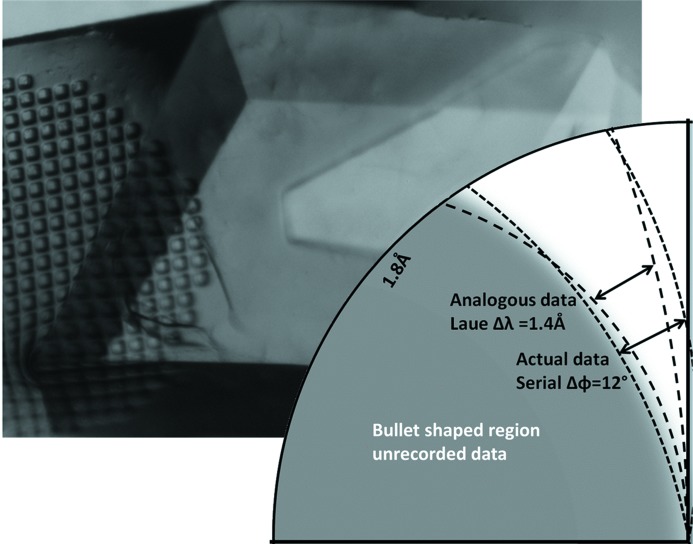
Strongly diffracting insulin crystals with side length ratios of ∼4:4:1 are easily grown over a very large range between 2000 µm and 4 µm. A view of one 300 µm crystal is shown here. The flat rhomboid face of these crystals frequently rests against the supporting micro-mesh, orienting the crystallographic *c* axis nearly parallel to the X-ray beam. Although the 5, 10 and 20 µm specimens used in this study were too small to orient visually, the diffraction data confirms a significant bullet-shaped region of un-measurable nodes adjacent to the *c* axis (inset). Flat insulin crystals preferentially orient with the *c* axis orthogonal to the support (*i.e*. into the X-ray beam). 6° librations from 100+ reciprocal space wedges all around the *c* axis guarantee that the volume of measurable data (dotted line) retains constant thickness around the X-ray beam. However, a bullet-shaped region of reciprocal space remains occluded (shaded region). The situation is analogous to a Laue case with λ_min_ ≃ 0.6 Å and λ_max_ ≃ 2.0 Å (dashed line). Fortunately, the missing data were readily available by tilting the micro-mesh about omega prior to the grid scan.

Removal of the mother liquor is crucial for optimizing the signal-to-noise of X-ray diffraction data. However, solvent minimization can also cause undesired side effects. One potential complication that can occur during solvent minimization is that the flat crystal faces may interact either with the surface of the data-collection medium (which may cause the crystallographic axis to always point the same way) or with neighboring crystals (which may cause the crystals to cluster into groups). The effect of these attractive forces was observed to depend on the shape and size of the protein crystals. We used crystals of insulin and lysozyme, which have very different crystal habits:

*Insulin*: Flat rhombohedral insulin crystals were grown by dissolving 0.025 g *Sus scrofa* (pig) insulin in crystallizing solution (2.0 ml 0.02 *M* HCl, 1.0 ml 0.20 *M* sodium citrate, 0.6 ml acetone, 0.2 ml H_2_O and 0.2 ml 0.12 *M* ZnSO_4_) at *T*_1_^soluble^ = 323 K and allowing the solution to cool slowly to *T*_2_^crystal^ = 293 K. The cooling rate determines the growth size for crystals (Table 1 ▶). Crystals of all sizes form a rhomboid with side length ratios of 4:4:1 (Fig. 2 ▶). By carefully controlling the cooling rate, our crystals were highly monodisperse with a side length variance of 40% of the mean (Soares *et al.*, 2003 ▶). Crystals were cryo-protected using 1.2 *M* trehalose or 30% *v*/*v* ethylene glycol. Specimens were deposited in a thin layer on micro-meshes, *in situ* micro-plates and on a movable crystal conveyor belt (Fig. 1 ▶).

*Lysozyme*: Cuboidal lysozyme crystals measuring 50 µm on each side were obtained by batch crystallization (120 mg ml^−1^ lysozyme dissolved in 0.1 *M* NaAc, pH 4.6 and 10% ethylene glycol, with 4% NaCl precipitant). Crystals were cryo-protected using 20% glycerol.

### 2.2. Data collection   

Serial crystallography data were obtained from crystals that resided on three types of data-collection media:

(i) On MiTeGen MicroMeshes™ (Fig. 1 ▶ *a*). Crystals mounted on micro-meshes had sizes between 5 µm and 50 µm. Data were obtained using a conventional goniometer.

(ii) On Greiner Bio-One™ micro-plates (Fig. 1 ▶ *b*). Plate data were obtained *in situ* from ∼50 µm crystals using a robotic G-Rob plate handler (NatX-ray LLC, San Diego, CA, USA) or using a conventional goniometer (Axford *et al.*, 2012 ▶). Goniometer-mounted plates were cut into 16 pieces, each of which was attached to a magnetic mount so that data could be obtained using a conventional goniometer.

(iii) On a movable conveyor belt system (Fig. 1 ▶ *c*). Data were obtained from crystals that were serially transferred to a goniometer-mounted conveyor belt system (Roessler *et al.*, 2013 ▶).

All of our small (<50 µm) specimens were serially interrogated by partitioning each crystal containing micro-mesh into 20 µm × 20 µm fields and scanning each field using the X-ray beam. Data were obtained from crystal-containing areas using either one 6° rotation or three 2° rotations (the beam size was matched to the expected crystal size). The unusually wide 6° data wedges were necessary so that the data from one crystal had sufficient reflections in common with the data from the next crystal for scaling^2^. Using much smaller wedges of data (>1°) would be feasible if the image-to-image matching process was automated, but the time needed to manually sort through thousands of single exposures to search for fortuitously oriented next-in-line images proved prohibitive. This scaling problem is mitigated when larger regions of reciprocal space are recorded from each micro-crystal.

Our specimens were exposed for a prolonged time interval to ensure that each 6° data wedge had a high *I*/σ(*I*). At X25, the nominal 2 × 10^11^ photons s^−1^ flux through 100 µm^2^ was *x*–*y* slit-shaped to form a mini-beam as small as 20 µm^2^. The flux density translates into ∼180 s needed to reach the Henderson dose limit (*D*_1/4_ = 10^7^ Gy), or 30 s per degree for a 6° wedge. A second data-collection pass with the micro-mesh rotated by 30–60° was sometimes needed (see §3.2), and the exposure time was reduced to allow for the additional data-collection sweep.

### 2.3. Merging data   

For insulin crystals, the *R*3 space group is polar so that re-indexing was frequently needed to stitch together the 6° data wedges from multiple crystals using *Scalepack* (Otwinowski & Minor, 2001 ▶). Our strategy was to pool a handful of compatible reduced images (about a dozen) by trial and error. Success required the coincidental presence of a sufficient number of simultaneously recorded reflections for each image to scale to the others. These scaled images then served as a ‘keystone’ to assemble each additional inbound image. Merging data from ∼100 crystals was straightforward when 3 × 2° contiguous wedges were measured. *HKL2000* had no difficulty indexing, scaling or merging each triad. Dealing with single 6° rotations was slightly more problematic (uncertainty in the crystal mosaicity impeded merging images that only shared a handful of simultaneously recorded reflections, so the ‘keystone’ images were more difficult to assemble). A more robust strategy for merging data in polar space groups was recently described (Brehm & Diederichs, 2014 ▶).

For comparison, data from differently sized crystals were segregated. For example, 10 µm and 20 µm crystals were separated. The high *R*_merge_ values are a consequence of merging data from ∼100 individual crystals into a single data set (Diederichs & Karplus, 1997 ▶). Relatively high *R*_merge_ values were tolerated by the structure determination software that we used and these initially high-*R*_merge_ values became increasingly insignificant as each data set supported the determination of a high-quality coordinate file. All six data sets supported a molecular-replacement solution using *MOLREP* (Vagin & Teplyakov, 1997 ▶) with a polyalanine search model (3rto and 1lyz, all residues changed to Ala) (Diamond, 1974 ▶). Each structure was completed and refined using *ARP/wARP* and *REFMAC* (Langer *et al.*, 2008 ▶; Murshudov *et al.*, 2011 ▶). Parameters for data collection, structure solution and refinement are shown in Table 2 ▶ for all six data sets from insulin crystals. The three data sets from lysozyme crystals are not shown. Other than completeness differences discussed later, there were no significant differences between the quality of data obtained from serial data collection using micro-meshes, *in situ* plates and the movable conveyor belt system.

## 3. Results   

**Figure 3:**
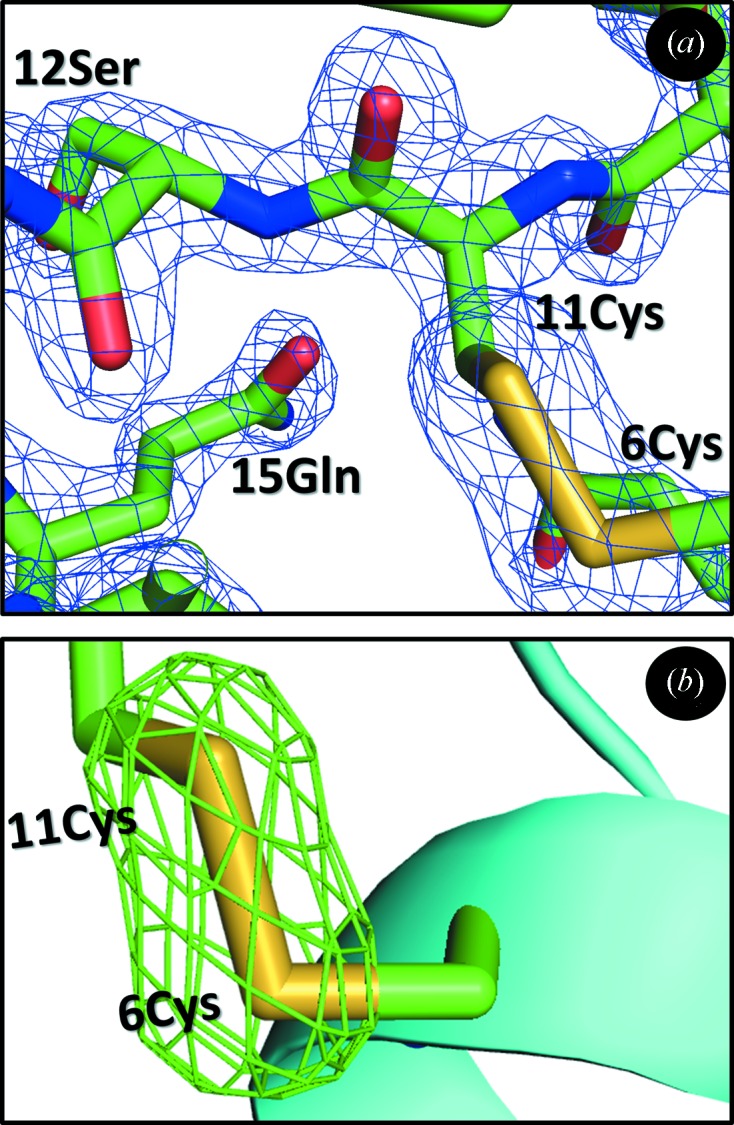
Crystals with a longest side length of 10 µm or more yielded molecular-replacement structures from polyalanine search models. (*a*) After refinement, the electron density from 192 different 20 µm crystals adequately fits the model (*R* = 22.3, *R*_free_ = 27.7). The region shown is in the vicinity of 6Cys–11Cys (2*F*_o_ − *F*_c_ contoured at 3σ). The disulfide bond shows no signs of breakage, because the high overall radiation dose was spread over many crystals. (*b*) A close-up view of the same region is shown with the refined model of the *in situ* plate mounted crystal (data merged from 88 crystals, *R* = 21.0, *R*_free_ = 26.3). A difference omit map is contoured at 2σ (the two cysteine sulfur atoms were omitted from the model). No evidence of damage to the disulfide bond is visible, even though the data were obtained at room temperature (where radiation damage should have been larger).

Data from small crystals mounted on micro-meshes were obtained at NSLS-X12b from 20 µm porcine insulin crystals in space group *R*3 scanned by a 30 µm × 30 µm beam, and at NSLS-X25 from 10 µm and 5 µm porcine insulin crystals scanned by a 20 µm × 20 µm beam (Table 2 ▶). Data from larger lysozyme and insulin crystals (>50 µm) were obtained at NSLS-X25 (micro-meshes), NSLS-X29 (*in situ* plates) and NSLS-X12c (conveyor belt). Usable data sets were assembled from 6° reciprocal space wedges measured either with a single 6° rotation or with three adjacent 2° rotations, as indicated in the table. Wedges were indexed and scaled using *HKL2000* and collated together using *Scalepack*. 5 µm crystals almost always produced overlapping diffraction patterns and the amount of indexable data was insufficient to build an atomic model. Crystals with a longest side length of 10 µm or more yielded high-quality structures (Fig. 3 ▶). The refined models show no evidence of radiation damage such as broken disulfide bonds.^3^

### 3.1. Solvent minimization   

**Figure 4:**
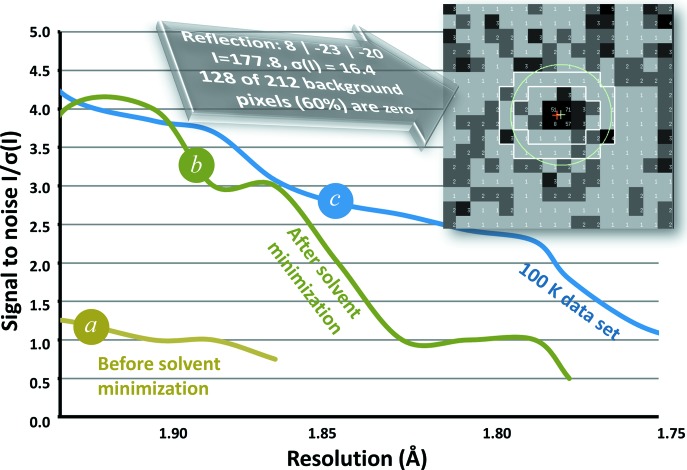
Solvent minimization. The signal-to-noise ratio [*I*/σ(*I*)] is plotted for the same thermolysin crystal at room temperature before solvent minimization (*a*), at room temperature after solvent minimization (*b*) and at 100 K (*c*). The room-temperature data sets consisted of 0.1° rotations with a duration of 0.2 s, with the X-ray beam attenuated by 97%. The 100 K data set consisted of 2.0° rotations with a duration of 2.0 s, using the full X-ray beam. The inset shows the 8 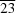 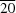 reflection after solvent minimization. The background integration box for this reflection had exactly zero observed counts for 60% of the pixels (so that the readout value was equal to the Pilatus 6M baseline of 1).

Fig. 4 ▶ compares the signal-to-noise ratio [*I*/σ(*I*)] for room-temperature X-ray data (0.2 s per 0.1° rotation with 97% attenuation of the X-ray beam) obtained from the same thermolysin crystal before and after the solvent minimization procedure (§2.1), as well as data from the same crystal after cryo-cooling in liquid nitrogen (2.0 s per 2.0° rotation with no attenuation). The solvent minimization greatly improved the quality of the X-ray diffraction data. The resolution limit (S/N > 1.0) was improved from 1.91 to 1.77 Å. The room-temperature data after solvent minimization have zero counts observed in most of the background pixels.

### 3.2. Preferential orientation   

The flat rhomboid habit of our insulin specimens preferentially orients the crystallographic *c* axis normal to the collection medium surface (Fig. 2 ▶). To simplify software code development, we initially scanned our grids with the X-ray beam traversing the flat face at a 90° angle. This meant that the *c* axis of the majority of the crystals was approximately parallel to the X-ray beam during the experiment. Although the axis of rotation was perpendicular to the X-ray beam, the 6° of rotation for each specimen was much less significant than the effect of sampling hundreds of crystals with random rotations around a fixed *c* axis. Consequently, our initial experiment sampled all possible rotations of crystals with the *c* axis approximately parallel to the beam, convoluted with a small 6° libration. This resulted in a very substantial ‘bullet-shaped’ region of unobtainable data (Table 2 ▶). The initial data sets resembled single-crystal Laue stills with λ_min_ ≃ 0.6 Å and λ_max_ ≃ 2.0 Å (Fig. 2 ▶, inset).

**Figure 5:**
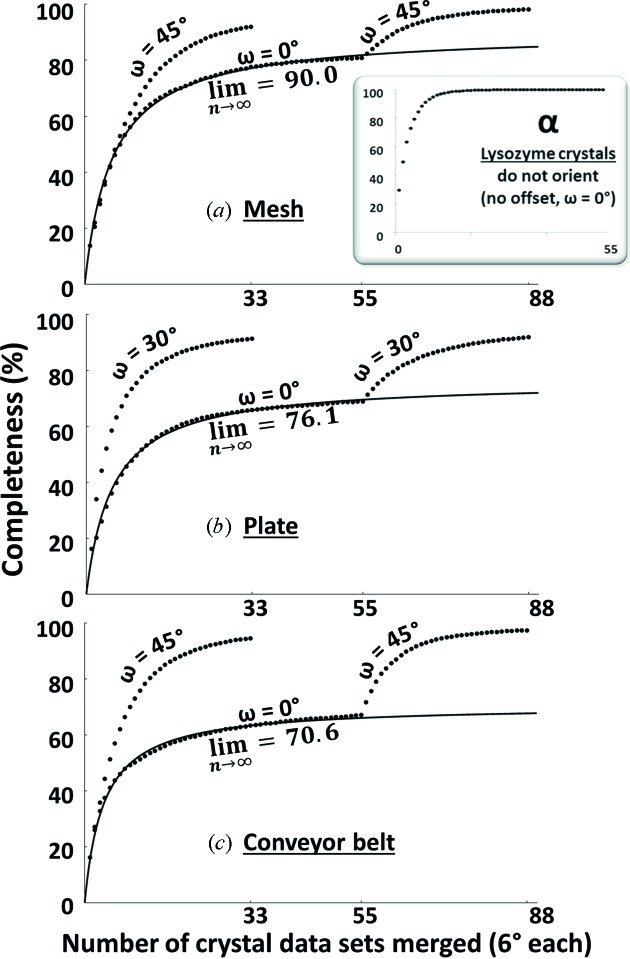
These graphs show completeness of the data from porcine insulin (percent) against the number of 6° data wedges that were merged together from crystals on micro-meshes (*a*), on *in situ* plates (*b*) and on a movable conveyor belt (*c*). Two plots are shown in each panel. The lower plot shows completeness as 55 × 6° data wedges are merged with the flat surface of the data-collection media normal to the X-ray beam, followed by 33 × 6° data wedges with the flat surface offset to the X-ray beam by 30° (plates) or 45° (micro-meshes and conveyor belt). In the upper plot the offset data wedges are merged by themselves. An asymptotic best fit shows that, without the offset, the completeness of the data approaches a limit value of less than 100%. In contrast, when offset data are included (either alone or in combination with the ‘flat’ data), 100% completeness is achieved. The data were smoothed by merging the 6° wedges in 100 random permutations, and averaging the results. The inset shows a similar calculation for 6° wedges of data from 55 lysozyme crystals (panel α). The lysozyme crystals do not appear to preferentially orient their crystallographic axes relative to the micro-mesh surface. The completeness of the merged data file rapidly approaches 100% regardless of whether the micro-mesh is presented normal to the X-ray beam or at an angle. The same results were observed with lysozyme crystals in Grainer *in situ* plates and on the conveyor belt (data not shown).

The completeness of each data set asymptotically approached a limit value that was less than 100%. The maximum completeness was calculated using a least-squares fit to equation (1),
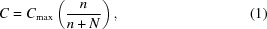
where *C* is the observed completeness, *C*_max_ is the limit completeness after addition of many data sets, *n* is the number of data sets merged, and *N* is a unit-less fitting parameter. The best values for *C*_max_ and *N* were calculated by least-squares fit. The limit completeness was approximately 90% when using micro-meshes, and even less for crystals on plates and on the conveyor belt (micro-mesh data have a higher limit completeness because the micro-meshes have a natural curvature which prevents the crystals from being exactly normal to the X-ray beam). To address the problem of limited completeness, data were obtained from a limited number of specimens after rotating the collection media by 30 to 60° (Fig. 5 ▶).

It is likely that the flat shape of insulin crystals plays a role in aligning the crystallographic *c* axis perpendicular to the surfaces on which the insulin crystals are deposited. In contrast, lysozyme crystals have a cuboidal habit with no extended planar surface. Consequently, lysozyme crystals were not observed to preferentially orient a crystallographic axis (Fig. 5 ▶, inset α).

### 3.3. Crystal clustering   

**Figure 6:**
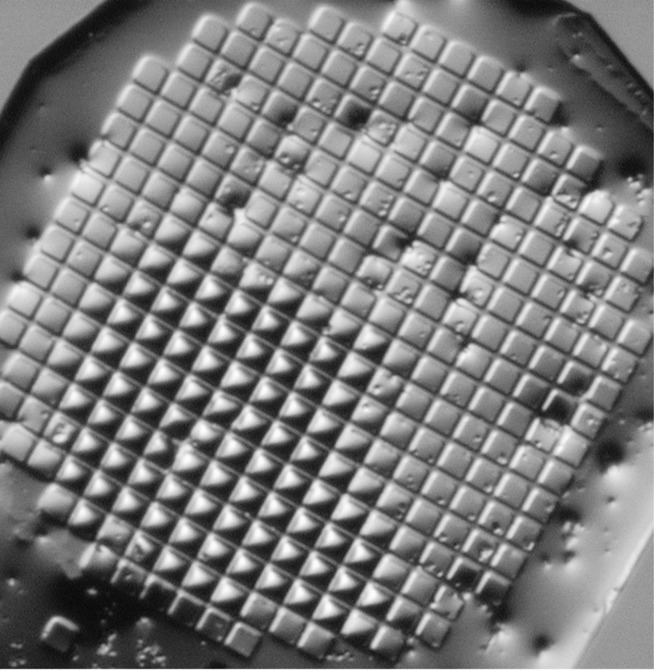
Careful minimization of mother liquor was an indispensable step to observe adequate signal-to-noise in the diffraction pattern. One undesired side effect is that solvent minimization induces crystals to aggregate into tight clusters of crystals that yield overlapping diffraction patterns. The 5 µm insulin crystals shown here diffracted to 2.3 Å, but no structure was forthcoming because of persistent overlap problems. Realistic molecular-replacement structures were readily obtained from 10 µm crystals and from 20 µm crystals.

The 5 µm crystals proved extremely difficult to index because virtually every rotation image was either blank or contained too many overlapping diffraction patterns. After examining many such images, we suspected that the distribution of 5 µm crystals within each micro-mesh could not be random. Close visual examination of several micro-meshes confirmed that all of our specimens aggregated into clusters during the mother liquor wicking procedure, and that this clustering was more pronounced with small crystals compared with large crystals. Fig. 6 ▶ shows one example of small insulin crystals that are highly clustered.

**Figure 7:**
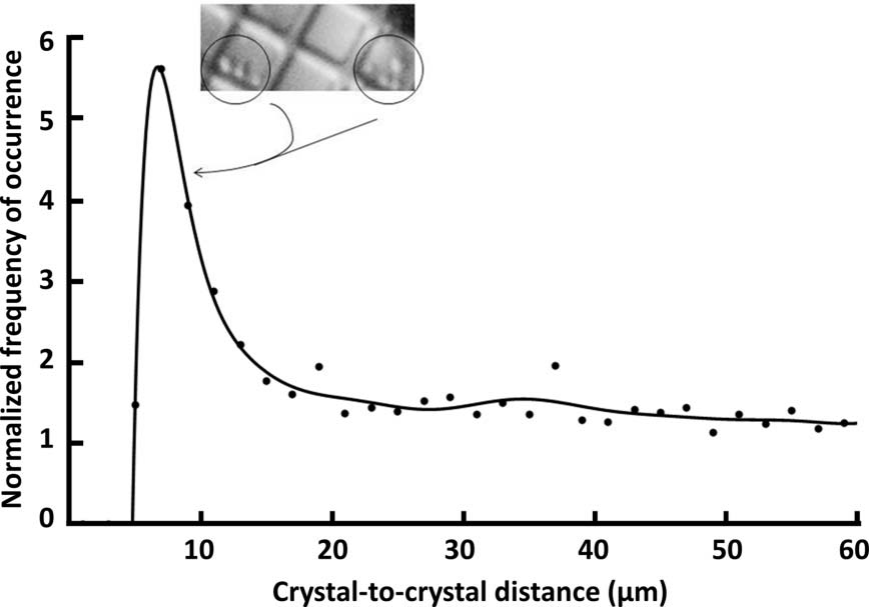
Severe clustering of insulin crystals made indexing difficult for 5 µm crystals (seen here on a micro-mesh with 20 µm openings). Frequently, our scanned images were either empty or had multiple overlapping diffraction patterns (inset). The attraction between crystals is predominantly short range. The *x*, *y* coordinates were determined for 450 crystals visible on a 700 µm micro-mesh. The distance between each crystal and every other crystal was calculated. The graph shows the relative probability of encountering neighbors as a function of distance (µm). The curve is normalized by dividing the experimental probability distribution by the probability distribution obtained from computer-generated random locations; since the graph is normalized, a random experimental distribution would have a flat probability ratio equal to unity. A polynomial best fit is also shown. The spike at 7 µm (slightly larger than the average crystal size) indicates that there are many crystals located adjacent to other crystals. We hypothesize that surface tension may exert a short-range attractive force that draws crystals together. The relative probability falls below unity for distances of 200 µm or more.

A statistical analysis of the *x*,*y* coordinates for 5, 10 and 20 µm crystals on separate micro-meshes confirmed this pronounced clustering phenomenon. Fig. 7 ▶ shows the normalized frequency (5 µm average crystal size) of observation for each crystal-to-crystal distance (the distance between crystals was defined as the distance between the crystal centers). The figure shows a pronounced peak at 7 µm, indicating that small insulin crystals are very likely to be located immediately adjacent to another crystal. The clustering effect occurs over short ranges only, so that larger crystal-to-crystal distances are not affected. We did not determine what force causes crystals to cluster over short distances, but we hypothesize that surface tension may play a role.

Grid scans using a 5 µm beam at APS-GM/CA substantially mitigated this specimen overlap problem because when the beam size is comparable with the specimen size the crystals are likely to be illuminated one at a time. Once each crystal in a cluster is indexed with a small beam, it would be feasible to collect simultaneous data on all of them using a larger beam. Software being developed by other groups to de-convolute overlapping diffraction patterns is another alternative solution.

## 4. Discussion   

We demonstrate that a workable data set can be assembled from 100+ narrow reciprocal-space wedges drawn from 6° rotations of randomly oriented micro-crystals (10–20 µm) that are serially exposed on a moving crystal conveyor belt (our method was analogous to recent serial crystallography applications performed on micro-meshes and *in situ* crystallization plates) (Ji *et al.*, 2009 ▶; Axford *et al.*, 2012 ▶). As has been reported by others, uncomfortably high *R*_merge_ values are a natural consequence of merging data from multiple specimens, but this does not deter a high-quality molecular model. Aggressive efforts to minimize the solvent envelope around protein crystals can reduce the solvent contribution to background and improve the signal-to-noise of the X-ray diffraction data. We report two ways in which aggressive solvent minimization can induce crystals to arrange themselves in ways that negatively impact the data. First, crystals can aggregate into clusters, causing the X-ray beam to illuminate either too many crystals or none at all. Second, crystals can preferentially orient relative to the flat surface on which they are deposited (such as the flat micro-mesh, the smooth plate bottom or the conveyor belt). Preferential orientation of a crystallographic axis may cause serial crystallography experiments to over-sample one region of reciprocal space while neglecting other regions of reciprocal space.

Insulin crystals were observed to preferentially orient with the crystallographic *c* axis normal to the surface that they are deposited on. In contrast, lysozyme crystals were not observed to preferentially orient. Preferentially oriented crystals may serially transit the X-ray beam aligned so that large regions of reciprocal space are unmeasurable. The effects of preferential orientation on completeness can be avoided by positioning the data-collection surface at an angle to the incident beam. Alternatively, a second data-collection pass may be used to reveal regions of reciprocal space that are forbidden when the support surface is initially normal to the X-ray beam. It is perhaps not surprising that flat insulin crystals would tend to lie on a planar surface, while the orientation of cuboidal lysozyme crystals would remain random. This intuitive understanding of the causes of preferential orientation may facilitate recognizing situations where the problem is likely to occur, and designing experiments from the outset to avoid unmeasured regions of reciprocal space.

Careful specimen preparation with attention to solvent minimization yielded signal-to-noise and resolution limits comparable with those obtained from large single crystals. However, wicking away the solvent increased the attraction between crystals. In the case of very small specimens (∼5 µm) the aggregation was crippling and clean single-crystal diffraction patterns were virtually unobtainable when probing with a 20 µm beam. Subsequent experimentation at APS-GM/CA confirmed that clustering can be overcome by using an X-ray beam with a size comparable with the specimen size. We are investigating software improvements to handle multiple overlapping diffraction patterns, as well as physical methods to immobilize specimens while excess solvent is removed (such as adding structure to the kapton belt on our conveyor crystal delivery system to help disperse crystal clusters).

## Figures and Tables

**Table 1:** Nucleation frequency for insulin is proportional to the cool down rate from *T*_1_^soluble^ = 323 to *T*_2_^crystal^ = 293K Careful temperature control yields highly mono-disperse crystal sizes. Sizes were determined by inspection of high-resolution pictures.

Cool down	Number measured	Average size (m)	Standard deviation (m)	Smallest size (m)	Largest size (m)
*T*_1_ *T*_2_ 10s	255	5.2	1.5	3	12
*T*_1_ *T*_2_ 1min	159	9.7	3.5	5	27
*T*_1_ *T*_2_ 10min	89	21.6	7.9	8	53
*T*_1_ *T*_2_ 120min	74	47.3	14.9	22	103

**Table 2:** Data were obtained from 5m, 10m, 20m and 50m insulin crystals (shown below) as well as from lysozyme crystals (not shown) Each full data set (one per column) was merged from data from many crystals. Data from some crystals were obtained while the crystal support (micro-mesh, *in situ* plate or conveyor belt) was located normal to the X-ray beam, termed ‘Crystals @ 0’. Data from other crystals were obtained with the crystal support at an angle to the beam, termed ‘Crystals (angled)’. The smallest (5m) specimens did not yield sufficient data to build an atomic structure. The 20m crystals were exposed with three rotations of 2 each using a 30m beam at NSLS-X12b. A single 6 rotation was recorded from each 10m crystal under a 20m beam at NSLS-X25. Data sets that yielded atomic models are summarized below. Molecular replacement with a polyalanine search model was trivial. The side chains easily settled into the correct conformations using the difference density from the initial model. The quality of the coordinate files is summarized below. RMS deviations from ideal for the final structures are shown.

	Mesh	Mesh	Mesh	Mesh	Plate	Belt
Crystal size (m)	10	20	20	50	50	50
X-ray source	X25	X12b	X12b	X25	X29	X12c
Beam size (m)	20	30	30	50	100	200
Surface	Mesh	Mesh	Mesh	Mesh	Plate	Belt
Strategy	1 6	1 6	3 2	3 2	3 2	3 2
Resolution ()	1.7	1.9	1.9	1.8	1.8	1.8
Crystal number	101	114	192	88	88	88
Crystals @ 0	66	95	118	55	55	55
Completeness (%)	79.3	81.4	81.9	80.8	68.9	67.1
Limit (%)	82.5	87.0	86.3	90.0	76.1	70.6
Crystals (angled)	35	19	74	33	33	33
Angle to beam	60	60	60	45	30	45
Completeness (%)	93.4	89.8	96.3	98.1	91.8	97.4
Limit (%)	100.0	100.0	100.0	100.0	99.7	100.0
*R*_merge_ (%)	15.0 (71.2)	14.2 (59.7)	9.8 (51.2)	20.6 (99.2)	29.9 (92.9)	26.3 (61.7)
*I*/(*I*)	19.8 (1.3)	16.5 (1.0)	14.5 (1.9)	18.0 (1.0)	18.2 (1.0)	13.4 (2.9)
Multiplicity	4.6	7.8	14.2	4.5	12.5	5.4
No. of reflections	8747	5881	6307	7736	7241	7681
*R*_initial_ (%)	41.4	44.2	39.8	43.3	39.5	39.6
*R*_work_ (%)	26.6	22.3	22.3	21.2	21.0	25.9
*R*_free_ (%)	28.5	25.2	27.7	28.0	26.3	33.0
RMS deviations						
Bond lengths ()	0.037	0.031	0.026	0.023	0.034	0.040
Bond angles ()	2.825	2.678	2.416	2.283	2.772	3.541

## Footnotes

1 Some crystals were mounted from their crystallization plates onto MiTeGen micro-meshes using an Echo 550 liquid-handling instrument (Labcyte Inc., Sunnyvale, CA, USA). The Echo 550 transfers crystals enveloped in a thin 2.5 nL blanket of mother liquid (this small volume of solvent minimizes the X-ray background). We postulated that acoustically mounted crystals might have the advantage of low solvent (reduced background noise) without the disadvantages (preferential orientation described in §3.2 and crystal clustering described in §3.3). However, we observed that acoustically mounted specimens clustered together despite low mounting volumes.

2 The strategy of recording a 6° wedge of data from each crystal (three images of 2° width) is not optimal for most purposes. In this study the exaggerated frame width was selected for two reasons. First, it is much more difficult to merge narrow wedges of data, particularly when the space group is polar. Second, at NSLS II the conveyor belt will deliver crystals to the very bright AMX X-ray beam very rapidly and the 20 Hz framing rate for the Pilatus 6M detector will not be able to keep up. For this reason we are interested in exploring how much of a data quality penalty is caused by measuring unusually wide rotations.

3 The disulfide bonds in insulin are highly susceptible to bond breakage induced by X-ray radiation (Helliwell, 1988 ▶).
